# Cannabinoids and the Endocannabinoid System in Early SARS-CoV-2 Infection and Long COVID-19—A Scoping Review

**DOI:** 10.3390/jcm13010227

**Published:** 2023-12-30

**Authors:** Cassidy Scott, Stefan Hall, Juan Zhou, Christian Lehmann

**Affiliations:** 1 Department of Anesthesia, Pain Management and Perioperative Medicine, Dalhousie University, Halifax, NS B3H 1X5, Canada; cassidy.scott@dal.ca (C.S.); juan.zhou@dal.ca (J.Z.); 2 Department of Physiology and Biophysics, Dalhousie University, Halifax, NS B3H 1X5, Canada; shall@dal.ca

**Keywords:** cannabidiol, cannabinoids, COVID-19, THC, viral infection

## Abstract

Coronavirus disease-19 (COVID-19) is a highly contagious illness caused by the SARS-CoV-2 virus. The clinical presentation of COVID-19 is variable, often including symptoms such as fever, cough, headache, fatigue, and an altered sense of smell and taste. Recently, post-acute “long” COVID-19 has emerged as a concern, with symptoms persisting beyond the acute infection. Vaccinations remain one of the most effective preventative methods against severe COVID-19 outcomes and the development of long-term COVID-19. However, individuals with underlying health conditions may not mount an adequate protective response to COVID-19 vaccines, increasing the likelihood of severe symptoms, hospitalization, and the development of long-term COVID-19 in high-risk populations. This review explores the potential therapeutic role of cannabinoids in limiting the susceptibility and severity of infection, both pre- and post-SARS-CoV-19 infection. Early in the SARS-CoV-19 infection, cannabinoids have been shown to prevent viral entry, mitigate oxidative stress, and alleviate the associated cytokine storm. Post-SARS-CoV-2 infection, cannabinoids have shown promise in treating symptoms associated with post-acute long COVID-19, including depression, anxiety, post-traumatic stress injury, insomnia, pain, and decreased appetite. While current research primarily focuses on potential treatments for the acute phase of COVID-19, there is a gap in research addressing therapeutics for the early and post-infectious phases. This review highlights the potential for future research to bridge this gap by investigating cannabinoids and the endocannabinoid system as a potential treatment strategy for both early and post-SARS-CoV-19 infection.

## 1. Introduction

Since its emergence in Wuhan, China, coronavirus disease-19 (COVID-19) caused by the severe acute respiratory syndrome coronavirus 2 (SARS-CoV-2) has spread globally, with over 770 million confirmed cases of COVID-19 reported by the World Health Organization as of September 2023 [1]. COVID-19 infections are classified as asymptomatic, pre-symptomatic, or symptomatic. Evidence from 113 studies completed across 17 countries showed that the viral load of SARS-CoV-2 peaks around the time of symptom onset or a few days after. 

Vaccinations are one of the most effective methods to prevent the severe outcomes of COVID-19. People diagnosed with COVID-19 after completing their primary vaccine series were significantly less likely to be hospitalized or die [2]. However, some patients with co-morbid conditions are at increased risk of an inadequate protective response to COVID-19 vaccines.

To date, the only FDA-approved agent for SARS-CoV-2 pre-exposure prophylaxis is Evusheld, which is composed of the anti-SARS-CoV-2 monoclonal antibodies tixagevimab and cilgavimab [3]. On-going clinical trials are investigating the pre-exposure use of other agents, including hydroxychloroquine, ivermectin, zinc, vitamin C, and vitamin D. However, studies have not demonstrated evidence of a reduced rate of acquiring infection and are not currently recommended for COVID-19 prophylaxis [4]. Effective COVID-19 prophylaxis is imperative for the health and wellbeing of high-risk populations and calls for further research into novel agents.

Recent evidence has shown that symptoms can remain after the clearance of an acute COVID-19 infection, in a condition referred to as post-acute COVID-19 syndrome, or more commonly, “long COVID-19.” Long COVID-19 is characterized by persistent and/or long-term complications that continue 3–4 weeks beyond the initial onset of acute COVID-19 symptoms [5]. Early reports found residual effects of the SARS-CoV-2 infection, including cognitive disturbances, arthralgia, headaches, chest pain, loss of taste/smell, cough, fatigue, and a decline in quality of life [6,7]. Various guidelines focus on managing long-term COVID-19, but as symptoms of this condition are heterogeneous and often attributed to underlying medical and psychiatric conditions, treatment plans mainly focus on symptom management and holistic support. More research is needed to understand the etiology of long-term COVID-19 and to provide insight on how to better treat this condition.

Cannabinoids have been shown to influence the course of infections both in vitro and in vivo. Cannabinoids act through cannabinoid receptor type 1 (CBR1) and cannabinoid receptor type 2 (CBR2). CBR1 is mainly expressed on neurons within the central nervous system (CNS), while CBR2 is expressed on cells of the immune system. Activation of CBR2 has effects on the host’s innate and adaptive immune responses, downregulating inflammation. Activation of CBR1 also has a role in host immune defense by inhibiting Ca^2+^ release, which in turn alters signal transduction pathways, the production of pro-inflammatory cytokines and reactive nitrogen intermediates, and apoptosis [8].

In recent years, the legalization of cannabis for both medical and recreational use has been approved in many countries [9]. With the legalization of cannabis being relatively new in many countries at the onset of the pandemic, several studies have been conducted to investigate changes in cannabis usage during the pandemic compared to the pre-pandemic period. A study by Statistics Canada found a 7% increase in cannabis use among the overall population, with 34% of previous cannabis consumers reporting an increase in their usage compared to the pre-pandemic period. Similar trends were observed in European countries such as France, Italy, and the Netherlands [10].

Given the reported increase in cannabis use during the pandemic and the potential interaction between endocannabinoid system (ECS) modulation and COVID-19 severity, this review aims to investigate the therapeutic potential of cannabinoids and the endocannabinoid system in mitigating the early stages of COVID-19 and the symptoms of long-term COVID-19.

## 2. Cannabinoids for Early SARS-CoV-2 Infection

### 2.1. Cannabinoids and Viral Entry

**Figure 1:**
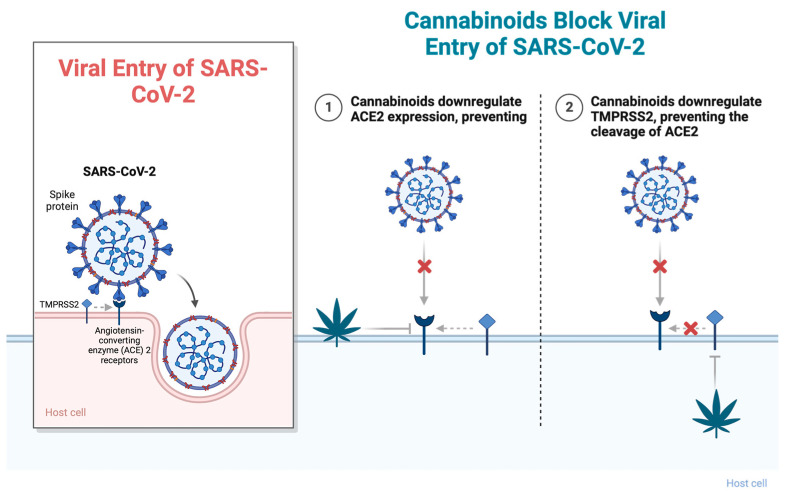
Cannabinoids block viral entry of SARS-CoV-2 through (1) downregulating ACE2 expression, preventing viral fusion, and (2) downregulating TMPRSS2, preventing the cleavage of ACE2. Abbreviations: ACE2, angiotensin-converting enzyme 2; TMPRSS2, type II transmembrane serine protease. Figure created with BioRender.com (accessed on 17 November 2023).

The viral spike protein of SARS-CoV-2 binds to the human angiotensin-converting enzyme 2 (ACE2) receptor, which is widely expressed in respiratory epithelia and vascular endothelium. The spike protein forms homotrimers that protrude from the SARS-CoV-2 surface and consist of an S1 and S2 subunit. The S1 subunit binds to ACE2 of host cells to initiate infection, while the S2 subunit mediates virus fusion with host cells and a transmembrane domain [10]. ACE2 is the main route for receptor-mediated entry of the virus into human hosts and is internalized upon binding. Studies by Xu et al. (2020) demonstrated that ACE2 is highly expressed on the mucosa of the oral cavity, suggesting that the oral cavity could be highly susceptible to SARS-CoV-2 infection [11]. Wang et al. (2020) studied over 800 new *Cannabis sativa* cultivars and tested their ability to downregulate ACE2 expression in SARS-CoV-2-targeted tissues. They found that high-cannabidiol (CBD) *C. sativa* extracts were able to decrease ACE2 protein levels in artificial 3D human models of oral, airway, and intestinal tissues. Additionally, they demonstrated that some extracts downregulated the type II transmembrane serine protease TMPRSS2 [11]. TMPRSS2 has been shown to bind and cleave the ACE2 receptor, leading to confirmational changes that allow the virus to fuse with the host membrane and enter the cell (Figure 1). Studies by Shulla et al. (2011) demonstrated that cells most susceptible to SARS-CoV infections are those in which ACE2 and type II transmembrane serine proteases (TTSPs) are simultaneously present [12]. Similarly, studies by van Breemen et al. (2022) found that cannabigerolic acid (CBGA) and cannabidiolic acid (CBDA) isolated from *C. sativa* prevented cell entry of SARS-CoV-2 into Vero E6 cells. CBGA and CBDA were equally effective against the SARS-CoV-2 alpha variant B.1.1.7 and the beta variant B.1.351 [12]. Small-molecule inhibitors of viral fusion have been effective against the human immunodeficiency virus, influenza, and paramyxovirus [13,14,15,16]. Cannabinoids may similarly have the potential to prevent infection by SARS-CoV-2.

### 2.2. Cannabinoids and Oxidative Stress

Research has demonstrated that patients suffering from viral infections, including respiratory diseases, show increased production of reactive oxygen species (ROS) [17]. Viral infections often disrupt the host’s redox homeostasis, increasing redox stress. Redox imbalance plays a key role in the pathogenesis and development of COVID-19, as an excess of ROS can damage cellular components, including DNA, proteins, and lipids, and alter immune functions and inflammatory responses [18,19,20]. Previous research has shown that oxidative stress contributes to the pathogenesis of respiratory viral infections, including influenza and respiratory syncytial virus (RSV) [17]. Similarly, increased oxidative stress in severe COVID-19 has been shown to contribute to inflammation, endothelial cell dysfunction, and thrombosis and can lead to multiorgan damage [21].

**Figure 2:**
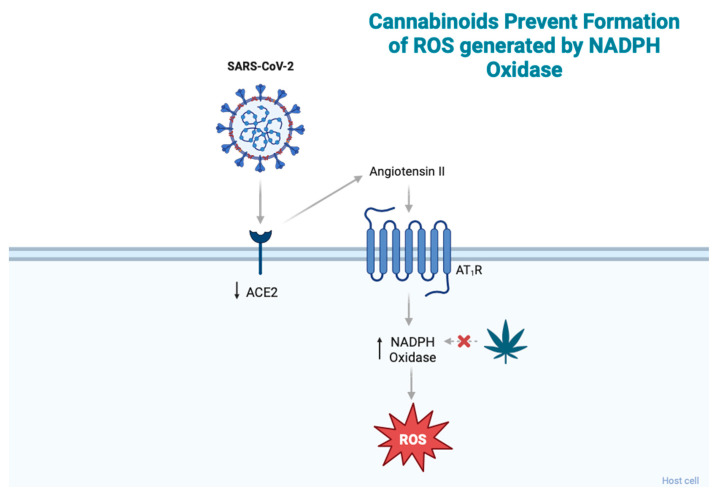
SARS-CoV-2 infection reduces ACE2 expression, leading to an accumulation of angiotensin II, which then binds AT_1_R and induces ROS production through NADPH oxidase. Cannabinoids have been shown to prevent the formation of ROS produced by NADPH. Abbreviations: ACE2, angiotensin-converting enzyme 2; ROS, reactive oxygen species; AT_1_R, angiotensin II type 1 receptor; NADPH, nicotinamide adenine dinucleotide phosphate. Figure created with BioRender.com.

ACE2, which is involved in viral entry, also plays a role in the induction of redox stress. SARS-CoV-2 infection reduces cell membrane ACE2 expression, leading to the accumulation of its primary substrate, angiotensin II (Ang II). Ang II binds the Ang II type 1 receptor (AT_1_R) and induces ROS production through nicotinamide adenine dinucleotide phosphate (NADPH) oxidase in the brain, vasculature, and kidneys (Figure 2) [20]. Thus, elevated Ang II causes increased AT_1_R activation, which contributes to increased ROS levels [20,22]. Cannabidiol (CBD), a phytocannabinoid that acts through the CBR2 receptor, has been shown to affect redox balance. Like other antioxidants, CBD interrupts free radical chain reactions, transforming free radicals into less active forms [23]. CBD has been shown to downregulate oxidative conditions by preventing the initiation of superoxide radicals produced by NAPDH oxidase and xanthine oxidase [24]. Studies by Pan et al. (2009) showed that CBD reduced oxidative conditions by preventing the formation of superoxide radicals generated by NADPH oxidase in a murine model of cisplatin-induced nephropathy [25].

An imbalance in the amount of oxidants and antioxidants has been reported in multiple medical conditions, including cancer, diabetes, obesity, infertility, neurodegenerative disorders, and lung diseases, where oxidative stress promotes tissue and cell damage [26]. Recently, cannabinoids have been studied as potential therapeutics for the oxidative stress associated with these conditions. Rajesh et al. (2022) studied the effects of pharmacological activation of CBR2 in a murine model of diabetes. They found that the selective CBR2 agonist JWH-133 attenuated diabetes-induced myocardial oxidative and nitrative stress. Furthermore, they observed that diabetic CBR2^-/-^ mice exhibited aggravated oxidative stress and inflammation compared with WT diabetic mice [27]. Similarly, Basha et al. (2016) demonstrated that administration of the CBR2 agonist beta-caryophyllene (BCP) significantly improved levels of antioxidant enzymes, decreased lipid peroxidative markers, and reversed levels of proinflammatory cytokine levels to near normal levels in STZ-induced diabetic mice, indicating a potential anti-inflammatory role of CBR2 agonists in preventing diabetes-induced oxidative stress [28]. Lastly, cannabinoids have been shown to play an antioxidative and neuroprotective role in neurodegenerative disorders such as Parkinson’s disease (PD). In a mouse model of PD, treatment with CBR1 agonists WIN55,212-2 and HU210 was found to increase the survival of nigrostriatal dopaminergic neurons in the striatum, suppress NOX and ROS production, and reduce pro-inflammatory cytokines [29]. Similarly, AEA was shown in vitro to protect hippocampal neurons from oxidative injury by decreasing ROS in a CBR1 receptor-mediated manner [30].

Together, these results suggest a potential protective role for cannabinoids in mitigating oxidative stress. Future studies should examine the antioxidative properties of cannabinoids as potential treatments for minimizing the pathogenesis and development of COVID-19.

### 2.3. Cannabinoids and the Cytokine Storm

During severe infection, SARS-CoV-2 suppresses innate anti-viral mechanisms while provoking a non-specific inflammatory response that is detrimental to the host. SARS-CoV-2 inhibits the production of type I interferons (IFN-Is), essential factors in early anti-viral defense, leading to unfettered viral replication. As more cells are infected, widespread induction of inflammatory cell death pathways causes a rapid increase in pro-inflammatory mediators that attract inflammatory cells, including neutrophils and monocytes, into lung tissue [5,6]. Studies have shown that a dysregulated and/or exaggerated cytokine response by SARS-CoV-2-infected cells could play a role in the pathogenesis and severity of COVID-19. For example, the associated endothelial barrier degradation and capillary leakage have been shown to contribute to alveolar cell damage, and inflammatory cytokine release, delayed neutrophil apoptosis, and NETosis have been shown to contribute to pulmonary thrombosis [7]. These mechanisms are in line with the observed clinical markers in COVID-19, including high expression of inflammatory cytokines (i.e., TNF-α/IL-6), elevated leukocyte and neutrophil counts, and an elevated neutrophil-to-lymphocyte ratio (NLR) [7,31]. Increased IL-6 is an early indicator of cytokine release syndrome, or the “cytokine storm.” A meta-analysis by Coomes et al. (2020) assessing current evidence in the field found that mean IL-6 concentrations were 2.9-fold higher in patients with complicated COVID-19 compared to patients with noncomplicated COVID-19, and elevated IL-6 was associated with adverse clinical outcomes [32].

Research has shown cannabinoids to be potent anti-inflammatory agents in the treatment of inflammatory diseases. Studies by Suryavanshi et al. (2022) found that CBD and THC significantly reduced levels of IL-6, IL-8, and TNF-α after LPS challenge in THP-1 macrophages and primary human bronchial epithelial cells (HBECs). Additionally, CBD attenuated the phosphorylation of nuclear factor-kB and inhibited the generation of oxidative stress [33]. Similarly, in a mouse model of acute respiratory distress syndrome (ARDS), treatment with THC led to a 100% survival rate, a reduction in the infiltration of immune cells into the lungs, and a reduction in the proinflammatory cytokines IFN-γ, IL-1β, IL-2, IL-6, and TNF-α. Similar trends were also observed for the chemokines CCL2, CCL5, and CXCL1 [34]. These results are promising, as ARDS is one of the major triggers of mortality associated with COVID-19 infections, and patients suffering from severe forms of COVID-19 have been shown to exhibit ARDS, cytokine storms, and pulmonary failure [35]. Comparably, studies by Khodadadi et al. (2020) found that administration of CBD downregulated levels of proinflammatory cytokines IL-6, IFN-γ, and TNF-α, reduced the number of infiltrating neutrophils and macrophages in the lungs, and improved clinical symptoms of Poly I:C-induced ARDS [36].

Results from these studies demonstrate the effectiveness of cannabinoids in alleviating cytokine storms and reducing the infiltration of inflammatory cells. Future studies should build upon these results, examining the potential effect of cannabinoids in treating ARDS and cytokine storms seen in COVID-19 patients.

## 3. Cannabinoids for Post-Acute “Long” COVID-19

### 3.1. Neuropsychiatric Sequela

Individuals suffering from COVID-19 have reported a range of psychiatric symptoms persisting or presenting months following the initial infection. A study conducted by Mazza et al. (2020) screened 402 adults one month following COVID-19 hospitalization for clinical signs of depression, anxiety, post-traumatic stress injury (PTSI), insomnia, and obsessive-compulsive (OC) symptomatology. They found that 56% of patients scored in the pathological range in at least one clinical dimension. Furthermore, patients with a positive previous psychiatric diagnosis showed increased scores on most measures [37]. Comparable results were found in a retrospective cohort study of 62,354 COVID-19 cases in the USA. The study found that in patients with no previous psychiatric history, a diagnosis of COVID-19 was associated with an increased incidence of a first psychiatric diagnosis in the following 14 to 90 days compared to following other health events, including influenza and other respiratory tract infections. Additionally, they found that the psychiatric effects of COVID-19 were broad but not uniform, with patients suffering from elevated rates of anxiety disorders, insomnia, and dementia [38].

The literature suggests that SARS-CoV-2 infection mediates dysregulation of the innate immune responses, leading to cytokine release syndrome with elevated pro-inflammatory cytokines and delayed IFN responses. Excess IL-6 and IL-1β with a simultaneous decrease in type I IFN levels has been reported to be positively correlated with disease severity and could drive neurological effects as a result of an altered blood–brain barrier (BBB) [39].

**Figure 3:**
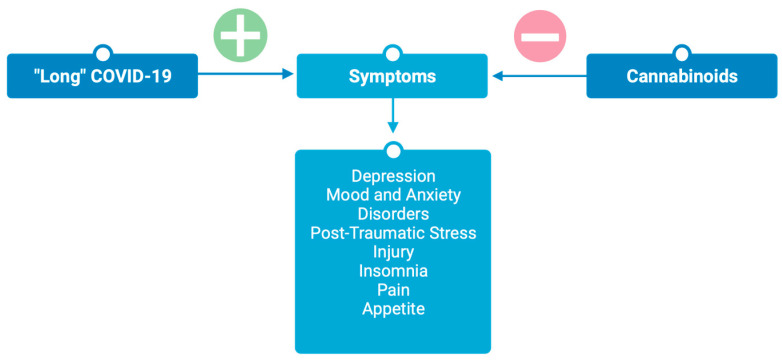
Cannabinoids attenuate symptoms associated with post-acute “long” COVID-19. Figure created with BioRender.com.

The endocannabinoid system is known to be involved in many neurological processes, including brain development, memory formation, learning, mood, anxiety, depression, analgesia, and drug addiction [40]. Modulation of the endocannabinoid system has been explored as a therapeutic for multiple neurological disorders, including Parkinson’s, Alzheimer’s, Huntington’s, multiple sclerosis, traumatic brain injury, stroke, epilepsy, anxiety, PTSI, and depression [41,42,43], indicating a potential role of ECS modulation as a therapeutic for persistent psychiatric symptoms associated with post-acute COVID-19 syndrome (Figure 3).

#### 3.1.1. Depression

Depressive symptoms and clinically significant depression are commonly reported among individuals suffering from post-COVID-19 syndrome. The prevalence of depressive symptoms in the United States increased over 3-fold during the COVID-19 pandemic compared to prior years [44]. Additionally, studies by Taquet et al. (2021) reported that the incidence of mood disorders in the 6 months following COVID-19 infection was significantly greater than after influenza or other respiratory tract infections [38]. Although it remains unknown whether depressive symptoms associated with post-COVID-19 syndrome are a consequence of the viral infection itself or related to the socio-economic outcomes of the pandemic, this increase in prevalence calls for novel approaches for the treatment of depression.

Pre-clinical studies using animal models have found anti-depressant-like responses to cannabis in behavioral tests, including the forced swim test (FST) and the tail suspension test (TST), two paradigms frequently used to evaluate the anti-depressant potential of agents. These tests are based on the principle that when an animal is exposed to a stressful and inescapable situation, it will first make efforts to escape but will eventually exhibit immobility that may be considered to reflect a measure of behavioral despair [45]. Studies by Zanelati et al. (2010) found that rats treated acutely or chronically (one single treatment or daily over 14 days) with 30 mg/kg of CBD had a reduction in immobility time and an increase in swimming time in the FST, compared to untreated controls [46]. Similarly, studies by Sartim et al. (2016) found that the administration of CBD (10–60 nmol) into the infralimbic and prelimbic subregions of rat brains significantly reduced immobility time in the FST [47]. Lastly, studies by Silote et al. (2021) found that administration of CBD produced anti-depressant-like effects in mice in the TST. However, significant effects were only seen in male mice and not females, indicating a potential sex-dependent effect of CBD as an anti-depressant [48].

Although, to date, no clinical trials have assessed the efficacy of cannabinoids for the treatment of depression, their use has been assessed in observational studies. Studies by Martin et al. (2021) found that medicinal cannabis use was associated with a significant decrease in depressive symptoms, an effect that was not observed in controls [49]. Similarly, Mangoo et al. (2022) examined the efficacy of 129 patients treated with cannabis-based medicinal products (CBMPs) for depression. Their results found that CBMP treatment was associated with a reduction in depression severity and an increase in health-related quality of life after 1, 3, and 6 months of treatment [50]. Lastly, an ongoing observational clinical trial (NCT04965740) is exploring the use of medicinal cannabis as a treatment for depression, PTSI, and anxiety in first responders and military personnel. The goal of this pilot trial is to collect data that will help inform and guide the development of a larger patient-oriented study and the design of a clinical program enhancing therapy treatments for these groups [51].

Human studies have found mixed effects of cannabinoid treatments on depression. Epidemiological studies have shown that non-medicinal or recreational use of cannabis, which is typically high in ∆9-THC, may be associated with an increased risk of developing depressive symptoms [52]. Studies on cannabis abuse as a risk factor for depression found that in participants with no baseline depressive symptoms, those with a diagnosis of cannabis abuse were four times more likely to develop depressive symptoms than those without a diagnosis of cannabis abuse [53]. Similar results were seen in studies by Lee et al. (2008), which found that heavy cannabis users, defined as those who smoke six or more cones of cannabis daily, were four times more likely to report moderate to severe depressive symptoms across three Aboriginal communities [54]. However, non-medical cannabis use may be used as a method of self-medication to cope with depressive episodes and may not be representative of its efficacy when used at a prescribed dosage. Furthermore, studies have shown synthetic forms of ∆9-THC to be effective anti-depressants at low doses and not at high doses, where the reverse effect of worsening of depressive symptoms was observed [55]. Non-medicinal cannabis is typically high in ∆9-THC, and when smoked, the amount of ∆9-THC delivered can vary from person to person. This may account for the observed discrepancies between epidemiological studies examining the effects of smoking cannabis and randomized control trials where the dosage of cannabinoids delivered is fixed.

#### 3.1.2. Mood and Anxiety Disorders

Mood and anxiety disorders have been reported in many patients suffering from post-acute COVID-19 syndrome. A cohort study conducted at Jin Yin-tan Hospital in China found that 23% of patients suffered from persistent symptoms of anxiety 6 months after symptom onset [56]. The endocannabinoid system is extensively distributed across the central nervous system (CNS) and plays a crucial role in the modulation of emotional responses, including fear, anxiety, and stress responses [57].

Pre-clinical and animal studies have shown evidence that supports the non-psychotropic cannabinoid, CBD, as a treatment for mood and anxiety disorders. Multiple studies assessing the effects of CBD in animal models of anxiety, including the elevated plus maze, the Vogel conflict test, and contextual fear conditioning, found CBD to have an anxiolytic effect at doses ranging from 1–10 mg/kg [58,59,60,61,62]. Furthermore, the effects of CBD have been studied on human experimental anxiety. Studies by Zuardi et al. (1993) found that CBD (300 mg) decreased anxiety following a simulated public speaking test [63]. Similarly, Bergamaschi et al. (2011) found that pre-treatment with CBD (600 mg) significantly reduced anxiety, cognitive impairment, and discomfort in speech performance in control subjects and patients suffering from untreated generalized social anxiety disorder following a simulation of the public speaking test [64]. Lastly, a recent review published by O’Sullivan et al. (2021) summarizes findings from clinical randomized control trials, observational studies, and case reports demonstrating the anxiolytic effects of CBD in treating patients with symptoms of anxiety, suffering from generalized anxiety disorder, sleep disorders, Crohn’s disease, depression, and PTSD [65].

#### 3.1.3. Post-Traumatic Stress Injury

Post-Traumatic Stress Injury (PTSI) is a neurological condition where individuals suffer from persistent, recurring memories of traumatic events and are unable to repress such memories. In a systemic review assessing the neuropsychiatric sequelae of COVID-19, PTSI has been reported as a persistent symptom associated with post-acute COVID-19 syndrome in 20 research articles, with PTSI ranging from 6.5% to 42.8% of the included patients [66].

Recently, there has been an increase in research on the therapeutic potential of cannabis and synthetic cannabinoids. Studies by Roitman et al. (2014) have demonstrated the efficacy of ∆9-THC, a phytocannabinoid CBR1 receptor agonist, in treating PTSI. Their results found that treatment twice a day with 5 mg of orally absorbable ∆9-THC significantly improved global symptom severity in ten outpatients with chronic PTSI [67]. Additionally, studies by Cameron et al. (2014) have assessed the efficacy of synthetic cannabinoids in treating PTSI. They found that daily treatment with nabilone (0.5–6.0 mg), a synthetic cannabinoid CBR1 receptor agonist, significantly improved PTSI symptoms and PTSI-associated insomnia in a population of mentally ill inmates [68]. Similarly, Fraser et al. (2009) reviewed the charts of 47 patients diagnosed with PTSI. These patients had been referred to the author’s private outpatient clinic and were treated nightly with nabilone at a starting dose of 0.5 mg. The dose was titrated up or down to effect, with an effective dose range of 0.2 mg to 4 mg. Their results found that 72% of patients experience total cessation or lessening of the severity of PTSI-associated nightmares, and some patients reported a reduction in daytime flashbacks [69].

#### 3.1.4. Insomnia

A one-year follow-up cohort study monitoring persistent symptoms in 303 patients who were diagnosed with COVID-19 reported a prevalence of sleep disorders in 47% of patients [70]. Recently, cannabinoids have gained acceptance in the medical community as a treatment for insomnia. In an animal model, activation of the CBR1 receptor through the administration of ∆9-THC has been shown to promote sleep, and these effects were blocked with the selective CBR1 antagonist SR141716A, suggesting that ∆9-THC is modulating sleep by the CBR1 receptors [71]. A systematic review and meta-analysis evaluating the efficacy of cannabinoids in the treatment of insomnia found favorable effects of cannabinoids on the Pittsburgh Sleep Quality Questionnaire, Insomnia Severity Index (ISI), and sleep latency test [72]. Similar results were seen by Walsh et al. (2020), who developed a sublingual cannabinoid extract (ZTL-101) containing 3 mg of ∆9-THC, 0.3 mg of cannabinol (CBN), and 0.15 mg of CBD to treat chronic insomnia. This was the first randomized double-blind placebo-controlled crossover trial assessing the use of cannabis-based drugs to treat chronic insomnia, and their results showed that ZTL-101 significantly improved ISI scores, total sleep time, and self-reported sleep latency [72]. Together, these results support further investigation into novel cannabinoid therapies for the treatment of insomnia.

### 3.2. Pain

Pain has been frequently reported as a lasting symptom, burdening patients suffering from post-acute COVID-19 syndrome. Studies by Sykes et al. (2020) reported myalgia as a lasting symptom in 51.5% of patients [73]. Similarly, Lombardo et al. (2021) found that 48% of patients experienced prevalent symptoms of pain 12 months following an acute COVID-19 diagnosis [70]. Multiple forms of pain have been associated with post-acute COVID-19 syndrome, including back pain, body pain, arthralgia, abdominal pain, and chest pain [74].

Pain and inflammation are part of the body’s physiological response to infection. Local vasodilation increases capillary permeability, and the release of pro-inflammatory mediators leads to the sensation of pain and hyperalgesia. Once the condition causing the damage is resolved, the associated pain and inflammation typically subside. However, in cases where the diseased condition and the associated pain and inflammation do not resolve, the inflammatory response progresses towards subacute or chronic inflammation, characterized by an overexpression of pro-inflammatory genes and a dysregulation of cellular signaling and barrier function [75]. Although the mechanisms causing long-term COVID-associated pain remain unclear, it has been suggested that the inflammatory response caused by the virus may affect the central and peripheral nervous systems, promoting the perpetuation of pain [73]. Additionally, it is likely that a portion of the associated pain experienced by previously hospitalized patients may be due to prolonged intubation and immobility, which often results in weakness, rapid deconditioning, and joint-related pain [76]. Chronic pain is a key factor affecting patients’ quality of life. Cannabinoid receptor modulation has been studied for the treatment of pain in various disease models, presenting as a potential novel approach to treating pain associated with post-acute COVID-19 syndrome.

Preclinical studies have demonstrated the potential anti-nociceptive activity of cannabinoid receptor agonists. Several studies examine these analgesic effects in models of healthy rodents subjected to painful experiences. Studies by Malan et al. (2002) found that the selective CBR2 agonist AM1241 produces anti-nociception in rats exposed to thermal stimuli. These effects were blocked with the CBR2 antagonist AM630 but not with the CBR1 antagonist AM251, demonstrating the CBR2-mediated analgesic activity [77]. Similarly, studies by Martin et al. (1998) found that systemic administration of the CBR agonists WIN55,212-2 and HU-210 produced anti-nociception in the rat tail-flick reflex. When administered together, both compounds significantly increased tail-flick latencies by over 50% [78]. Lastly, studies by Harris et al. (2019) examined the analgesic effects of *C. sativa* extracts in a rat model of acute pain. They found that the full *C. sativa* extract, the extract without terpenes, and the isolated ∆9-THC all produced dose-dependent increases in hotplate and tail-flick latencies [79].

In addition to acute animal models of pain, studies have also shown the potential of endocannabinoid receptor modulation for the treatment of pain in humans. Data from clinical trials assessing synthetic and plant-derived cannabis-based medicines for the treatment of chronic neuropathic pain have shown promising results. A double-blind, randomized placebo-controlled crossover trial by Wilsey et al. (2013) examined the efficacy of vaporized cannabis for the treatment of neuropathic pain. Their results found that 57% of participants exposed to a low dose (1.29% ∆9-THC) and 61% of participants exposed to a medium dose (3.53% ∆9-THC) reported a 30% reduction in pain intensity. There was no significant difference between the two active dose groups. However, an analgesic response to vaporized cannabis was seen compared to placebo [80]. In a follow-up study, Wilsey et al. (2016) examined the effects of vaporized cannabis containing either placebo, 2.9%, or 6.7% ∆9-THC in patients with neuropathic pain related to disease or injury of the spinal cord. Administration of ∆9-THC at both active doses showed a significant decrease in pain intensity compared to placebo on an 11-point numerical pain rating scale. Furthermore, vaporized cannabis positively and significantly improved all of the measured multidimensional pain descriptors, except itching, on the Neuropathic Pain Scale (NPS) [81].

In addition to vaporized cannabis products, recent research has been interested in isolating the major components of cannabis responsible for the analgesic properties and treating patients with such medicinal extracts. A case study by Romeyke and Westfal (2022) studied the analgesic effects of cannabis extract THC/CBD 10:10 mg in a patient suffering from multilocular chronic acute exacerbated pain syndrome. This study found that the administration of THC/CBD, stepped from 0.5 mL/day to 1 mL/day over the course of 15 days, resulted in a decrease in pain intensity from 8/10 to 4/10 on the visual analogue scale and an improvement in the quality of sleep [82].

Lastly, a randomized, double-blind, placebo-controlled clinical trial by Nurmikko et al. (2007) studied the efficacy of Sativex^®^ oromucosal spray (2.7 mg ∆9-THC: 2.5 mg CBD) on neuropathic pain. They found a significant reduction in pain intensity scores in patients receiving Sativex^®^ compared to placebo, as well as improvements in NPS score, sleep, dynamic allodynia, punctate allodynia, and Patient’s Global Impression of Change score. Currently, Sativex^®^ is approved as an adjunctive treatment for symptomatic relief of spasticity in patients with MS in Europe, New Zealand, and Canada [83].

Cannabinoids have also been tested for the treatment of painful conditions, including headaches, back, chest, and abdominal pain, all of which are symptoms commonly experienced by patients suffering from long-term COVID-19. A randomized, double-blind, active-controlled, crossover study compared nabilone (0.5 mg/day) to ibuprofen (400 mg/day) for treating medication-overuse headaches. This study found nabilone more effective than ibuprofen in reducing pain intensity and daily analgesic consumption [84]. Furthermore, a study by Pinsger et al. (2006) investigated the efficacy of nabilone as an add-on treatment in patients with chronic pain. It was found that nabilone (0.5 to 1 mg/day) decreased the average headache intensity and increased the number of days without headaches. Nabilone also decreased the average back pain intensity and increased quality of life scores [85]. Similarly, a retrospective study by Ueberall et al. (2021) compared the efficacy of nabiximol oromucosal spray vs long-acting opioids for the treatment of neuropathic back pain. Both treatments showed a significant improvement in pain symptoms compared to baseline, with all between-group differences significantly favoring cannabinoids [86]. Oral cannabinoids such as dronabonial have also shown promise in treating chest pain. Studies by Malik et al. (2017) found treatment with dronabinol significantly increased pain threshold and reduced pain intensity in patients suffering from noncardiac chest pain [87]. Lastly, cannabinoids have commonly been used for symptom relief of abdominal pain associated with inflammatory bowel disorders (IBDs) [88,89,90,91]. Studies by Storr et al. (2014) assessed cannabis usage in patients with IBDs. Their results found that 17.6% of IBD patients used cannabis for symptom relief, 83.9% of whom reported that cannabis improved abdominal pain [92]. Similarly, Naftali et al. (2011) performed a retrospective, observational study to describe the effects of cannabis use on patients suffering from Crohn’s disease. They found that cannabis significantly improved scores on the Harvey Bradshaw index, which assesses abdominal pain, general well-being, number of liquid stools, abdominal mass, and complications [93]. The authors later completed the first randomized, double-blind, placebo-controlled trial treating Crohn’s disease patients with marijuana cigarettes (300 mg/day). Patients in the cannabis group reported significantly less abdominal pain compared to placebo. Furthermore, two patients who treated severe abdominal pain with opiates stopped opiates during the study [94].

Inflammatory pain is a major clinical problem affecting patients’ quality of life. As CBR2s are primarily expressed in immune cells, including macrophages, microglial cells, lymphoid myeloids, and mast cells, they present a potential target for treating inflammatory pain. Studies by Yuill et al. (2017) found that the selective CBR2 agonist (JWH-133) produced dose-dependent anti-nociception effects in both the acute and inflammatory phases of the formalin test. Furthermore, administration of the CBR2 antagonist SR2 blocked the anti-nociception effects of JWH-133, demonstrating that these effects are mediated through activation of CBR2 [95]. Similarly, studies by Hsieh et al. (2011) assessed the efficacy of CBR2 agonists in the complete Freund’s adjuvant (CFA) model of chronic inflammatory pain. They found that CFA administration into the central footpad leads to the upregulation of CBR2. Treatment with the CBR2 selective agonist A-836339 produced a significant reversal of the CFA-induced decrease in paw withdrawal latency. Systemic administration of the CBR2 selective antagonist SR144528 reversed the A-836339 anti-hyperalgesia effect while the CBR1 selective antagonist did not, indicating that the effects of A-836339 are mediated through activation of the CBR2 [96]. In humans, cannabinoids have shown efficacy in reducing the inflammatory pain associated with MS and arthritis. Studies by Rog et al. (2005) found that administration of an oromucosal spray containing 2.7 mg of ∆9-THC and 2.5 mg of CBD significantly reduced the mean intensity of pain and sleep disturbances compared to placebo [97]. Similarly, Svendsen et al. (2004) demonstrated that the oral synthetic cannabinoid dronabinol significantly lowered pain intensity scores compared to placebo [98]. Lastly, studies by Blake et al. (2006) found that Sativex^®^ oromucosal spray significantly improved pain on movement, pain at rest, and quality of life scores compared to placebo in patients suffering from pain caused by rheumatoid arthritis [99].

Results from the studies discussed above demonstrate evidence supporting the analgesic effects of cannabinoids for the treatment of pain. Although further research is required and should focus on exploring the benefits of cannabinoids in controlled clinical trials, these results support the notion that cannabinoids should be considered as a treatment option in the management of pain associated with long COVID-19.

### 3.3. Appetite

The World Health Organization (WHO) defines the most common symptoms of post-acute COVID-19 to include fatigue, shortness of breath, cognitive dysfunction, persistent cough, and pain. However, the definition also includes gastrointestinal (GI) issues such as altered sense of smell and taste, diarrhea, and constipation. In a systematic review of 50 studies by Choudhury et al. (2022), it was found that GI symptoms as part of long-term COVID-19 occurred in 22% of patients. These symptoms included loss of taste, loss of appetite, abdominal pain, nausea and vomiting, and diarrhea [100]. Although the mechanisms behind the GI manifestations occurring following the COVID-19 infection are not completely understood, they are thought to be related to the increased expression of ACE-2 in the small bowel mucosa. Prolonged viral shedding in fecal samples has been reported for nearly five weeks after the patients’ respiratory samples tested negative for SARS-CoV-2 RNA and could be related to some of the GI symptoms associated with long COVID-19 [101]. Although the GI manifestations of long COVID-19 are not well recognized, they are frequently reported in patients suffering from post-acute long COVID-19 and are likely to result in work-related absences and a decrease in quality of life. Recently, cannabinoids have been shown to reduce chemotherapy-induced nausea and vomiting and to improve appetite in those with cancer and HIV/AIDS [102,103]. As such, the endocannabinoid system presents a potential pharmacological target to modify eating behaviors and responsiveness to food in those suffering from GI manifestations associated with post-acute COVID-19.

In an observation study by Weng et al. (2021) analyzing symptoms of post-acute long COVID-19 12 weeks following the initial infection, nausea and vomiting were reported in 18% and 9% of patients, respectively [104]. The efficacy of cannabis-based medications has been tested for chemotherapy-induced nausea and vomiting in adults with cancer. Clinical studies by Meiri et al. (2007) found that oral administration of dronabinol (2.5 mg) showed similar efficacy in reducing the intensity of nausea and number of vomiting episodes as ondansetron, a medication commonly administered to prevent nausea and vomiting in cancer patients. Furthermore, dronabinol performed significantly better at treating nausea intensity than placebo [105]. A systematic review by Smith et al. (2015) found that cannabinoids were highly effective anti-emetics. Patients who received cannabinoids were five times more likely to report a complete absence of vomiting and three times more likely to report a complete absence of both nausea and vomiting compared to those who received placebo. Additionally, there was no significant difference between cannabinoids and the anti-emetic prochlorperazine in the proportion of patients reporting no nausea, no vomiting, or complete absence of the two [106]. Similar results were reported in a systematic review by Rocha et al. (2008), who found that dronabinol had better acute anti-emetic efficacy than conventional anti-emetic drugs (prochlorperazine, chlorpromazine, domperidone, haloperidol, alizapride, and metoclopramide) on cancer patients treated with chemotherapeutic agents. Patients taking dronabinol reported fewer vomiting episodes and a greater improvement in the severity of nausea compared to conventional anti-emetics [107].

In addition to the reported nausea and vomiting, olfactory dysfunctions following viral infections have profound impacts on quality of life as they alter the typical eating experience and how food tastes and smells. In addition to loss of taste/smell, parosmia and dysgeusia have been reported as symptoms of post-acute COVID-19. These symptoms are related to a loss of appetite, with patients reporting a fear of eating due to food tasting and smelling unpleasant [108].

The endocannabinoid system is known to be involved in appetite, eating behavior, and body weight regulation. Endocannabinoids acting at the CBR1 stimulate appetite, partly through interactions with orexigenic and anorexigenic signals. The endocannabinoids anandamide and 2-AG have been shown to promote feeding when administered into hypothalamic nuclei and into the shell of the nucleus accumbens, regions firmly associated with eating motivation [109]. Preclinical studies have shown that the exogenous cannabinoid ∆9-THC and the endocannabinoid anandamide stimulate eating in rats. Williams et al. (2002) found that administration of ∆9-THC (0.56 to 1.8 mg/kg) produced hyperphagia and increased consumption of a high-fat diet when administered to rats. Furthermore, the hyperphagia and preference for a high-fat diet produced by ∆9-THC administration were blocked by the CBR1 inverse agonist SR-141716 [110]. In a clinical study assessing the use of dronabinol as a treatment for AIDS-related anorexia, Beal et al. (1995 found that dronabinol (2.5 mg, twice daily) significantly increased appetite above baseline. Furthermore, weight remained stable in dronabinol patients, while patients receiving placebo had a mean loss of 0.4 kg [111]. Currently, dronabinol and nabilone are approved by the FDA for HIV/AIDS-induced loss of appetite and for nausea and vomiting associated with cancer chemotherapy in adult patients who failed conventional anti-emetics [112].

## 4. Therapeutic Applications

### 4.1. Routes of Administration

Throughout this review, many routes of administration are discussed, including oral oils, capsules, and solutions (e.g., Dronabinol and Nabilone), oromucosal sprays (e.g., Sativex^®^ and Nabiximols), and smoked or vaporized cannabis. In the studies discussed above, routes of administration vary, and little is known to what degree results can be generalized to other routes of administration. It is well known that the effects of medical cannabis vary by route of administration in terms of onset of action, desired benefits, and side effects, but there is a lack of research comparing the medicinal benefits across administration routes. Although smoking and vaporizing cannabis are often preferred by patients, as they provide the fastest onset of effects, it is possible that the potential benefits of cannabinoid treatments are outweighed by the negative respiratory health consequences associated with smoking. Studies have suggested that the use of cannabis vaporizers is associated with fewer respiratory symptoms than smoking cannabis, as they do not heat marijuana to the point of combustion [113]. However, limited research has been conducted comparing the long-term respiratory effects of the two methods of administration, which is often complicated by the co-morbidity of cannabis and cigarette smoking. Nevertheless, long-term smoke inhalation through smoking or vaporizers is likely to reduce respiratory health and is not suggested as a treatment for a respiratory virus. Alternative routes of administration, such as oral oils, capsules, and oromucosal sprays, have the potential to reduce the respiratory health risks associated with smoking cannabis and are known to have longer-lasting effects but slower onset.

To date, there is a broad range of cannabis-based products available in many countries with a prescription or through authorized retailers where cannabis is legal. However, very little is known about the efficacy, dose, or side effects of commonly used, commercially available cannabis products. These products often contain a combination of cannabinoids and hundreds of other compounds with poorly understood effects. Synthetic cannabinoid products have similar effects to natural cannabis but show differences in their selectivity, potency, and function. Although their dose can be carefully titrated, these drugs bind cannabinoid receptors with a higher affinity than cannabis, are more potent, and have the potential for adverse effects. Future research is needed to compare the various routes of administration, forms, and combinations of cannabinoids.

### 4.2. Limitations and Recommendations

Cannabis and cannabinoid-based drugs have shown promise in preventing viral entry, acting as an anti-inflammatory agent, and improving many symptoms associated with post-acute SARS-CoV-2 infections. However, these results do not come without limitations. Firstly, the potential for the use of cannabis and cannabinoid-based drugs is limited to adults. Although limited studies have been conducted assessing its therapeutic use in youth and early adulthood, research has shown that cannabis use in these populations may alter neurodevelopment and increase the risk of psychotic symptoms [114]. Additionally, cannabinoid products, like many other treatments, should not be used by individuals who are pregnant, planning to become pregnant, or breastfeeding.

Secondly, there is a lack of standardization for commercially available cannabis products. Levels of cannabinoids can vary significantly depending on the strain, growth conditions, and preparation methods. This variability makes it difficult to ensure consistent dosing. Furthermore, individuals may react differently to cannabis. It is important to note that many factors (i.e., metabolism, health conditions, genetics) may influence how a patient reacts to cannabis, and like most therapies, cannabinoid therapies should be individualized for the patient. Each patient should have a comprehensive assessment and risk–benefit discussion to avoid potential complications. A conservative dosing and titration protocol beginning with a lower and slower dose may be preferred to assess tolerability. When prescribing cannabis, clinicians should also consider the chemical phenotype of the cannabis plant, as different strains contain different concentrations and combinations of cannabinoids. Medicinal cannabis can be bred to have a specific chemical phenotype that is high in the desired cannabinoid. Cannabis containing cannabinoids activating the CBR1, such as ∆9-THC, can cause unwanted psychoactive effects that need to be considered even at low doses. These central effects can cause a decrease in alertness, prevent the patient from operating heavy machinery, and may have occupational and recreational hazards. Prescribing clinicians may consider dosing CBR1 agonists in the evenings to prevent potential issues with work and day-to-day activities.

Lastly, there is a lack of well-controlled, double-blind, randomized clinical trials assessing the medicinal benefits of cannabis and cannabinoid-based products. Studies are generally short in duration and have small sample sizes. More clinical trials are needed, as well as additional research on the pharmacology, pharmacokinetics, and mechanism of action of medical cannabis, in order to develop more targeted treatments.

## 5. Conclusions

Endocannabinoid system modulation presents as a potential target in early and post-acute SARS-CoV-2 infections. The existing literature suggests that the ECS plays a crucial role in regulating the immune system and inflammatory processes. Cannabinoids have the potential to be used as a preventive approach to limiting the susceptibility and severity of COVID-19 infections by preventing viral entry, mitigating oxidative stress, and alleviating the associated cytokine storm. Furthermore, cannabis and cannabinoid-based drugs have shown promise in treating many symptoms associated with post-acute COVID-19 syndrome. Although ECS modulation holds potential as a treatment strategy, it is important to acknowledge the limitations of this scoping review. The majority of studies supporting ECS modulation as a treatment strategy have been conducted in contexts other than COVID-19, and therefore extrapolation of these findings to SARS-CoV-2 infections requires caution. To fully understand the efficacy and safety of cannabinoid-based drugs in the context of COVID-19, further research is required. Clinical trials and well-designed studies are necessary to assess the underlying mechanisms, determine optimal dosages and dosing schedules, and investigate the safety and potential side effects associated with ECS modulation in the context of viral infections. Therefore, despite the promising outlook, a comprehensive understanding of these aspects is crucial for establishing the therapeutic potential of cannabinoids and ECS modulation on the onset of COVID-19 and lingering symptoms associated with long COVID-19.

## Data Availability

No new data were created or analyzed in this study. Data sharing is not applicable to this article.
